# Sentinel Lymph Node in Endometrial Hyperplasia: State of the Art and Future Perspectives

**DOI:** 10.3390/cancers17050776

**Published:** 2025-02-24

**Authors:** Valentina Billone, Lina De Paola, Eleonora Conti, Letizia Borsellino, Zoltan Kozinszky, Pierluigi Giampaolino, Andrea Suranyi, Luigi Della Corte, Alessandra Andrisani, Gaspare Cucinella, Susanna Marinelli, Giuseppe Gullo

**Affiliations:** 1 Department of Obstetrics and Gynaecology, AOOR Villa Sofia—Cervello, University of Palermo, 90146 Palermo, Italy; valentina.billone@gmail.com (V.B.); elexconti97@gmail.com (E.C.); letiziaborsellino93@gmail.com (L.B.); gasparecucinella1@gmail.com (G.C.); 2 Department of Anatomical, Histological, Forensic and Orthopedic Sciences, Sapienza University of Rome, 00161 Rome, Italy; lina.depaola@uniroma1.it; 3 Department of Obstetrics and Gynecology, Albert Szent-Gyorgyi Medical School, University of Szeged, Semmelweis u. 1, H-6725 Szeged, Hungary; kozinszky@gmail.com (Z.K.); gaspar-suranyi.andrea@med.u-szeged.hu (A.S.); 4 Capio Specialized Center for Gynecology, Postgången 53, 171 45 Solna, Sweden; 5 Department of Public Health, University of Naples Federico II, 80131 Naples, Italy; pgiampaolino@gmail.com; 6 Department of Neuroscience, Reproductive Sciences and Dentistry, School of Medicine, University of Naples Federico II, 80131 Naples, Italy; luigi.dellacorte@unina.it; 7 Department of Women’s and Children’s Health, Gynaecologic and Obstetrics Clinic, University of Padua, 35122 Padua, Italy; alessandra.andrisani@unipd.it; 8 School of Law, Polytechnic University of Marche, 60121 Ancona, Italy; susanna.marinelli@tiscali.it

**Keywords:** endometrial hyperplasia, sentinel lymph node, sentinel lymph node mapping, pelvic lymphadenectomy, endometrial carcinoma

## Abstract

**Background/Objectives**: Endometrial hyperplasia is a uterine pathology characterized by the abnormal proliferation of endometrial glands, resulting in an increased gland-to-stroma ratio. Complex atypical hyperplasia represents the primary precursor to endometrial cancer. Given the high risk of progression to endometrial adenocarcinoma, the accurate diagnosis and classification of endometrial hyperplasia are crucial. Since the treatment for atypical endometrial hyperplasia is the same as that for early-stage endometrial cancer (i.e., total hysterectomy and bilateral salpingo-oophorectomy), researchers have questioned whether sentinel lymph node mapping could also have a prognostic role in atypical endometrial hyperplasia. **Methods**: A literature search was conducted in PubMed and Scopus from 2014 to 2025. Of the 65 papers found, 31 relevant articles were selected based on inclusion criteria, focusing on sentinel lymph node staging in patients with atypical endometrial hyperplasia. This review aims to assess whether sentinel lymph node mapping can be routinely used for prognostic and therapeutic purposes in clinical practice. **Results**: Sentinel lymph node biopsy represents a promising diagnostic technique for patients with atypical endometrial hyperplasia, reducing the need for invasive procedures and postoperative risks. However, its application requires advanced surgical skills and access to specific technologies, raising ethical and financial concerns, while future studies could improve patient selection and the reliability of the procedure through technological innovations. **Conclusions**: Sentinel lymph node biopsy is a safe and effective method for staging early-stage endometrial cancer and atypical hyperplasia, with low metastasis rates, but future research should focus on identifying patients who would benefit most from this procedure, considering its costs and required expertise.

## Simple Summary

This research aims to evaluate the role of sentinel lymph node biopsy (SNB) in patients with atypical endometrial hyperplasia and early-stage endometrial carcinoma. The authors seek to determine whether SNB can provide accurate cancer staging while reducing the need for more invasive procedures, such as pelvic lymphadenectomy, and minimizing associated risks and healthcare costs. This study explores how SNB could improve patient outcomes by offering a less invasive and potentially more effective diagnostic method. The findings could lead to more informed clinical decisions, optimizing patient care and resource utilization. For the research community, this study may stimulate the further exploration of non-invasive techniques for cancer staging, influencing future guidelines and practices in gynecological oncology.

## 1. Introduction

Endometrial hyperplasia is a uterine pathology characterized by the abnormal proliferation of endometrial glands, resulting in an increased gland-to-stroma ratio. From a histological standpoint, it can be classified as either simple or complex (with or without atypia) [1]. Complex atypical hyperplasia represents the primary precursor to endometrial cancer, particularly the endometrioid histotype. Clinically, it often manifests as abnormal or postmenopausal uterine bleeding, as 10–20% of postmenopausal uterine bleeding cases are caused by endometrial hyperplasia or cancer [2].

The underlying cause of endometrial hyperplasia is believed to be chronic and excessive estrogen stimulation without adequate progesterone compensation. Consequently, the primary risk factors for endometrial hyperplasia are the same as those for type I endometrial adenocarcinoma: obesity, advanced age, and conditions that cause hyperestrogenism, such as polycystic ovary syndrome (PCOS), delayed menopause, and nulliparity [1,3].

Given the high risk of progression to endometrial adenocarcinoma, the accurate diagnosis and classification of endometrial hyperplasia are crucial. However, this is not always straightforward due to the heterogeneity of its architectural features. Indeed, numerous studies have shown that up to 50% of patients who underwent preoperative endometrial biopsy with a diagnosis of atypical endometrial hyperplasia were subsequently found to have endometrial cancer on definitive histological examination [4].

This has led to questions about the optimal therapeutic strategy for endometrial hyperplasia. For simple hyperplasia, medical therapy with oral or intrauterine progestins may be considered. However, for complex atypical hyperplasia, the treatment of choice is the same as that for early-stage endometrial cancer: total hysterectomy with bilateral salpingo-oophorectomy. In selected cases where the patient desires a child, fertility-sparing treatment with levonorgestrel-releasing intrauterine devices or oral progestins may be considered, along with lifestyle modifications such as weight loss and improved glycemic control [5,6].

Since the treatment for atypical endometrial hyperplasia is the same as that for early-stage endometrial cancer (i.e., total hysterectomy and bilateral salpingo-oophorectomy), researchers have questioned whether sentinel lymph node mapping could also have a prognostic role in atypical endometrial hyperplasia. Complex atypical hyperplasia (CAH) of the endometrium is a precursor lesion for endometrial cancer [7]. Up to 42% of cases of CAH diagnosed with endometrial biopsy are found to harbor endometrial cancer on hysterectomy specimens [7]. Guidelines for the management of complex endometrial hyperplasia lack clear direction, and practice patterns vary from no staging to intraoperative endometrial assessment and conventional lymph node sampling. This lack of consistency ultimately results in some endometrial cancer cases being unstaged, undertreated, or overtreated [8]. This review aims to analyze the role of sentinel lymph node mapping in the nodal staging of endometrial hyperplasia to better understand whether it can be routinely used in clinical practice for both prognostic and therapeutic purposes, while also identifying potential gaps.

## 2. Materials and Methods

A literature search was conducted regarding lymph node staging in patients with atypical endometrial hyperplasia. A comprehensive search was performed in PubMed and Scopus covering the period from 2014 to 2025. The following keywords were searched: “atypical endometrial hyperplasia AND sentinel lymph node” and “endometrial hyperplasia AND pelvic lymphadenectomy”.

**Figure 1:**
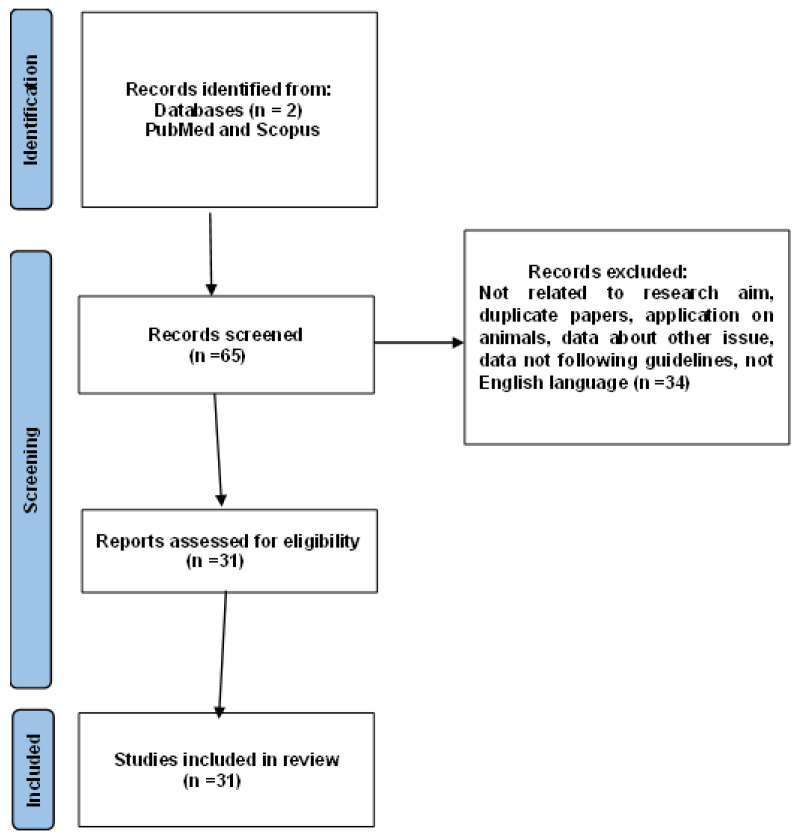
PRISMA flow chart.

We found 65 papers, and only those in English were included. Initially, the authors reviewed the titles and abstracts of all the papers, and if a study appeared potentially relevant, the full article was assessed. Inclusion criteria were articles relevant to the research topic, documents involving only human data, and reviews or articles with empirical data (Figure 1).

Ultimately, 31 articles were selected concerning the role of sentinel lymph node staging in patients with an initial diagnosis of atypical endometrial hyperplasia.

This review aims to analyze the role of sentinel lymph node mapping in the nodal staging of endometrial hyperplasia to better understand whether it can be routinely used in clinical practice for both prognostic and therapeutic purposes, while also identifying potential gaps.

## 3. Results

The studies analyzed in this literature review include cohorts of patients with a preoperative diagnosis of atypical endometrial hyperplasia, endometrial intraepithelial carcinoma, and early-stage endometrial carcinoma [4,9,10,11,12,13].

Touhami et al. analyzed the concept of a “borderline lesion” termed “atypical hyperplasia, cannot exclude well-differentiated carcinoma” as an intermediate category between AH and well-differentiated carcinoma [4]. The authors divided the spectrum of architectural complexity on endometrial biopsy specimens into three groups based on the associated risk of myoinvasion in final pathology: “complex atypical hyperplasia”, “complex atypical hyperplasia, cannot exclude well-differentiated adenocarcinoma” (borderline), and “well-differentiated adenocarcinoma” [4].

Most patients underwent radical hysterectomy, bilateral adnexectomy, and mono/bilateral sentinel lymph node mapping using Indocyanine Green or by systematic mono/bilateral pelvic lymphadenectomy using laparoscopic and robotic surgery techniques.

In some studies, patients underwent either sentinel node mapping or systematic pelvic lymphadenectomy, and the different impacts of the techniques on postoperative outcomes and of variations in efficacy on postoperative diagnosis were studied [4,9,10,12,14]. In other studies, some patients underwent only hysterectomy without a sentinel lymph node study [8,11,14,15].

The studies showed that positive lymph nodes at definitive histological diagnosis appeared in patients with a definitive histological diagnosis of endometrial carcinoma. In the study by Touhami et al., 3.3% of patients in the entire cohort (4/120) had lymph node involvement, representing 6.25% (4/64) of patients with EC at final pathological examination. When considering the initial group of patients with a preoperative diagnosis of “AH-C”, 8% (4/50) had EC with positive lymph nodes at final pathology. Interestingly, in the group of patients with EC with a preoperative diagnosis of “AH only”, none had lymph node metastases (0/31), whereas in the group of patients with EC with a preoperative diagnosis of “AH-C”, 4/33 (12.1%) had lymph node metastases [4].

In the study by Rosati et al., lymph node metastases were identified in 7.6% of patients with concomitant endometrial cancer who underwent lymph node assessment with at least unilateral mapping. Of the 12 sentinel lymph node metastases, 75.0% were micrometastases, 16.7% were macrometastases, and 8.3% were isolated tumor cells [15].

The studies also show that sentinel lymph node mapping appears to be safe; as shown in Table 1, there is no difference in terms of blood loss, it does not increase the operative time, it detects a small number of occult lymph node metastases for patients with tumor upstaging, and it provides additional staging information [10].

## 4. Discussion

In our research, sentinel lymph node biopsy (SNB) represents an important diagnostic tool, not only for confirmed cases of endometrial carcinoma but also for early forms such as atypical endometrial hyperplasia. In these cases, sentinel lymph node biopsy can aid in the evaluation of pathology, making it clearer and more accurate [16]. This, of course, positively impacts the patient’s clinical history, potentially allowing the avoidance of more extensive pelvic lymphadenectomy, thereby reducing both the healthcare-related costs of surgical interventions and the clinical risks associated with more destructive procedures [17]. However, it is important to note that atypical endometrial hyperplasia (AH) is not cancer by itself, and only a small percentage of AH cases are associated with early-stage endometrial carcinoma. In these cases, the likelihood of positive lymph nodes is low.

Therefore, while SNB may be useful in identifying potential lymph node metastases in confirmed or upstaged cases of carcinoma, the real challenge lies in identifying patients with atypical hyperplasia who may develop carcinoma or who might benefit from SNB in terms of altering management, particularly in the context of adjuvant therapy. If the pathological evaluation is accurate, the likelihood of lymph node metastasis in patients with isolated endometrial hyperplasia should theoretically be negligible [18,19,20,21]. To identify endometrial carcinoma associated with atypical hyperplasia, it would be useful to examine the endometrial biopsy, adopting not only a thorough histological examination, preferably performed by a specialist in the field, but also immunohistochemical methods or molecular analyses. Through the combination of multiple techniques, the ability to identify endometrial carcinoma associated with atypical hyperplasia could be improved, leading to better therapeutic decisions. Compared to other more invasive procedures like total pelvic lymphadenectomy, this technique offers the advantage of being easier to perform as it is less invasive and could also be more acceptable to patients [18,19,20]. Additionally, as previously mentioned, the risk of postoperative complications such as lymphedema, temporary or permanent nerve damage, or infections could be much lower [12,22,23].

Naturally, all these positive aspects also reflect the medico-legal perspective [24,25,26]. Every intervention is subject to potential complications, and the more surgical difficulty or destructiveness increases, the higher the surgical risk becomes, along with the potential for biological harm to the patient. This could lead to numerous legal disputes against health facility or medical professionals, as happens in many other branches of medicine across various fields. Healthcare professionals would therefore be held accountable for personal injury, and the decision to immediately perform total lymphadenectomy rather than sentinel lymph node biopsy could also be challenged. In this case, informed consent assumes a legally crucial importance that cannot be overlooked [27,28]. The patient must always be adequately informed, as in many other areas, of their condition and the possible diagnostic and therapeutic procedures available, specifying in each case the different potential complications. A failure to do so could lead to a legal dispute based on the violation of the patient’s right to self-determination, if they were unable to freely choose whether to undergo a certain procedure due to insufficient information. Moreover, it must always be considered that this procedure may give false negatives, failing to identify at-risk cases, especially in more specific scenarios. In such cases, it would then be necessary to perform subsequent total lymphadenectomy, which would nullify the advantages of sentinel lymph node biopsy. The patient should be properly informed of this as well. This type of situation and decision not only inevitably impacts the physical condition of the patient who has to undergo a surgical procedure but also has psychological repercussions that can be comparable to actual traumas, similar to those associated with a wide range of pathological conditions [29,30].

However, it is important to note that this procedure is certainly not an easily and ubiquitously executable one, as it requires a high level of surgical experience and access to the appropriate technologies, which may not be available in all centers. The procedure therefore brings with it ethical issues that the scientific community and policymakers should address [31,32,33]. Alongside these ethical issues, another similar problem is represented by the costs of the procedure [34,35]. While this technique reduces costs relative to more invasive interventions, the costs of the necessary technologies for SNB, such as radioactive tracking or colored tracers [36,37], and the training of specialized personnel must also be considered [38].

Finally, it is mandatory to discuss future prospects for the use of sentinel lymph node biopsy in endometrial hyperplasia. Future studies may lead to the development of predictive models to identify patients with endometrial hyperplasia who are more likely to develop endometrial carcinoma and lymph node metastases, thus improving the selection of patients for sentinel lymph node biopsy [39]. Furthermore, technological innovation, such as the use of advanced imaging techniques, including artificial intelligence [40,41], now applicable across all areas of medicine, or robotic surgery [42,43,44,45], could improve the reliability of sentinel lymph node biopsy by reducing false negatives and diagnostic errors.

## 5. Conclusions

Sentinel node biopsy (SNB) can be safely performed in patients with precursor lesions and early-stage EC without notably extending surgical times or increasing postoperative morbidity.

The rate of SLN metastases in patients with preoperative atypical hyperplasia/endometrial intraepithelial neoplasia is low. Sentinel lymph node studies in patients with a preoperative diagnosis of endometrial hyperplasia are useful in intercepting lymph node metastases in cases of cancer upstaging, i.e., in cases where the definitive histological examination shows a picture of endometrial carcinoma. Additionally, genetic and immunological tests could play a crucial role in identifying patients with atypical endometrial hyperplasia, helping to better identify those at risk of upstaging and enhancing treatment personalization.

In conclusion, future research should focus on which patients may benefit most from an SLN procedure, considering the additional costs and expertise required to perform this procedure.

## Figures and Tables

**Table 1:** Differences between analyzed studies.

Ref.	Number of Patients	Preoperative Histological Diagnosis	Lymph Node Staging	Postoperative Histological Diagnosis	Positive Lymph Nodes on Definitive Histological Examination	Postoperative Outcomes
[12]	102	Atypical endometrial hyperplasia (n = 20) and early-stage endometrial cancer (n = 82)	Sentinel node biopsy (n = 96)/sentinel node biopsy and pelvic lymphadenectomy (n = 6)	Atypical endometrial hyperplasia (n = 9) Endometrioid carcinoma (n = 90) Uterine serous carcinoma (n = 2) Adenosquamous endometrial cancer (n = 1)	3; 3.6% rate of positive SNBs was found in patients with EC	C *
[8]	113	Complex atypical hyperplasia (n = 74)/complex atypical hyperplasia bordering on cancer or unable to rule out cancer (n = 39)	Sentinel node sampling (n = 69), no sentinel node biopsy (n = 44)	No hyperplasia (n = 20) Complex atypical hyperplasia (n = 41) Endometrioid adenocarcinoma (n = 52)	1 ITC	C
[10]	221	EIN, complex atypical hyperplasia, and hyperplasia bordering on carcinoma	SLN mapping/excision (n = 161)	Endometrioid carcinoma (n = 98) Adenosarcoma (n = 1) CAH (n = 35) CAH bordering on carcinoma (n = 15) Atypical hyperplasia (n = 50) Benign (n = 22)	3	C
[14]	10,266	Complex atypical endometrial hyperplasia	Sentinel lymph node mapping in 620 (6.0%), lymph node dissection in 538 (5.2%), and no lymphatic evaluation in 9108	NA *	NA	C
[11]	10,217	Atypical hyperplasia/endometrial intraepithelial neoplasia and endometrial cancer	1044 in SLN group and 9173 in the non-nodal assessment group	NA	1.6%, 7 SLN	C
[4]	120	70 patients had diagnosis of “AH only” and 50 had preoperative diagnosis of “AH-C”	SLN mapping followed by pelvic lymphadenectomy	64/120 (53.3%) patients found to have EC on final pathology: 58 stage IA, 3 IB, and 3 IIIC1. 30/70 (44.3%) with AH and 33/50 (66%) with AH-C had EC on final pathology	4/120 had lymph node involvement. In patients with EC with preoperative diagnosis of “AH”, none had lymph node metastasis (0/31), 12.1% (4/33) in patients with AH-C	NA
[15]	460	Atypical endometrial hyperplasia	192 received standard surgical management (no SLN) and 268 underwent sentinel lymph node biopsy	47.2% of patients updated to endometrial cancer on final histopathological examination	Lymph node metastases were identified in 7.6% of patients with concurrent endometrial cancer who underwent nodal assessment	C
[9]	49,698	Endometrial hyperplasia	2847 (5.7%) patients had lymph node evaluation at time of hysterectomy	NA	NA	NA

* C: comparable; NA: not applicable.

## Data Availability

Data are available upon request from the corresponding author.
